# Mutations in the Arabidopsis *AtMRS2-11*/*AtMGT10*/*VAR5* Gene Cause Leaf Reticulation

**DOI:** 10.3389/fpls.2017.02007

**Published:** 2017-11-27

**Authors:** Shuang Liang, Yafei Qi, Jun Zhao, Yuanfeng Li, Rui Wang, Jingxia Shao, Xiayan Liu, Lijun An, Fei Yu

**Affiliations:** State Key Laboratory of Crop Stress Biology for Arid Areas and College of Life Sciences, Northwest A&F University, Yangling, China

**Keywords:** AtMRS2-11/AtMGT10/VAR5, chloroplast development, leaf variegation, Mg^2+^ transporters, chloroplast envelope

## Abstract

In higher plants, the development of functional chloroplasts is essential for photosynthesis and many other physiological processes. With a long-term goal of elucidating the genetic regulation of chloroplast development, we identified two allelic leaf variegation mutants, *variegated5-1* (*var5-1*) and *var5-2*. Both mutants showed a distinct leaf reticulation phenotype of yellow paraveinal regions and green interveinal regions, and the leaf reticulation phenotype correlated with photosynthetic defects. Through the identification of mutation sites in the two mutant alleles and the molecular complementation, we confirmed that *VAR5* encodes a CorA family of Mg^2+^ transporters also known as AtMRS2-11/AtMGT10. Using protoplast transient expression and biochemical fractionation assays, we demonstrated that AtMRS2-11/AtMGT10/VAR5 likely localizes to the chloroplast envelope. Moreover, we established that AtMRS2-11/AtMGT10/VAR5 forms large molecular weight complexes in the chloroplast and the sizes of these complexes clearly exceed those of their bacterial counterparts, suggesting the compositions of CorA Mg^2+^ transporter complex is different between the chloroplast and bacteria. Our findings indicate that AtMRS2-11/AtMGT10/VAR5 plays an important role in the tissue specific regulation of chloroplast development.

## Introduction

Magnesium ion (Mg^2+^) is one of the most abundant divalent cations in cells and is involved in myriads of essential metabolic processes (Maguire and Cowan, 2002). The electrically charged nature of Mg^2+^ necessitates the presence of membrane transporters to facilitate efficient Mg^2+^ trafficking across biological membranes. The functions and regulations of these transporters ensure the maintenance of Mg^2+^ homeostasis in different sub-cellular compartments in cells (Shaul, 2002).

Magnesium ion transport systems were most extensively studied in prokaryotes, and several types of putative Mg^2+^ transporters, including CorA, MgtE, and P-type ATPase Mgt, have been identified in bacteria (Smith and Maguire, 1995; Moomaw and Maguire, 2008). In eukaryotic cells, the CorA family Mg^2+^ transporter was identified in yeast mitochondria as MRS2 and is involved in group II intron splicing (Bui et al., 1999). Typical CorA Mg^2+^ transporters contain two C-terminal transmembrane domains and in between a conserved GMN motif as a Mg^2+^ binding site (Lunin et al., 2006; Pfoh et al., 2012). Genes coding for putative Mg^2+^ transporters are also present in plant genomes and the most extensively studied Mg^2+^ transporters in plants are the CorA family of transporters (Gardner, 2003). In the model system *Arabidopsis thaliana*, there are at least 10 genes encoding putative CorA Mg^2+^ transporters and they have been designated as encoding AtMRS2 or magnesium transport (AtMGT) (Schock et al., 2000; Li et al., 2001). Functionally, Arabidopsis CorA Mg^2+^ transporters were able to complement bacterial or yeast mutants that are defective in Mg^2+^ transport, suggesting that they are bona fide Mg^2+^ transporters (Schock et al., 2000; Li et al., 2001, 2008; Drummond et al., 2006; Mao et al., 2008; Chen et al., 2009; Gebert et al., 2009). Consistent with the important roles of Mg^2+^ in biological systems, *AtMRS2*/*AtMGT* genes have been shown to be important for several cellular processes. For example, AtMRS2-6/AtMGT5 and AtMRS2-2/AtMGT9 have been shown to be involved in pollen development (Li et al., 2008; Chen et al., 2009). Moreover, attempts to obtain insertional knock-out mutants of several *AtMRS2*/*AtMGT* genes have not yielded homozygous mutants for several genes including *AtMRS2-2*/*AtMGT9*, *AtMRS2-6*/*AtMGT5*, and *AtMRS2-11*/*AtMGT10*, suggesting these genes may be essential genes in Arabidopsis (Li et al., 2008; Chen et al., 2009; Gebert et al., 2009). Consistent with their membrane protein identities, these transporters are targeted to different sub-cellular compartments and are presumably responsible for Mg^2+^ homeostasis in various organelles. For example, AtMRS2-10/AtMGT1 and AtMRS2-4/AtMGT6 are plasma membrane Mg^2+^ transporters (Li et al., 2001; Mao et al., 2014). AtMRS2-6/AtMGT5 was shown to be targeted to mitochondria (Li et al., 2008). AtMRS2-7/AtMGT7 has been suggested to be located in the endoplasmic reticulum (Gebert et al., 2009). AtMRS2-1/AtMGT2 and AtMRS2-5/AtMGT3 are targeted to the tonoplast and involved in Mg^2+^ partition in leaf mesophyll cell vacuoles (Conn et al., 2011). In addition to the CorA Mg^2+^ transporters, other types of putative Mg^2+^ transporters have also been identified in plants including the AtMHX H^+^/Mg^2+^ transporter (Shaul et al., 1999;Shaul, 2002).

The chloroplast is the organelle for photosynthesis and many other essential processes in plants (Pogson and Albrecht, 2011). Mg^2+^ also plays critical roles in the chloroplast. For example, in the light reactions, Mg^2+^ is an integral part of photosynthetic pigment chlorophylls. In carbon reactions, it is known that Mg^2+^ can regulate the activities of ribulose 1,5-bisphosphate reductase/oxygenase (rubisco) and many other enzymes. Thus, it is not surprising that Mg^2+^ transporters are necessary in the chloroplast to facilitate the transport of the ion and to regulate chloroplast Mg^2+^ homeostasis. Of the AtMRS2/AtMGT family of Mg^2+^ transporters, AtMRS2-11/AtMGT10 is predicted to be targeted to the chloroplast based on the presence of a N-terminal chloroplast transit peptide (Drummond et al., 2006).

Plant variegation mutants generally refer to mutants that display differential leaf color in plant organs, especially in leaves (Yu et al., 2007). In variegation mutants defective in nuclear genes for chloroplast proteins, differential chlorophyll accumulation/chloroplast development can arise from a uniform mutant genetic background, and these mutants have long attracted attention because the mechanisms of variegation are instrumental in our understanding of chloroplast development. Among variegation mutants, one unique group of leaf variegation mutants is the reticulate mutants (Yu et al., 2007). These mutants usually show a differential chlorophyll accumulation/chloroplast development between paraveinal regions and interveinal regions, thus giving rise to a “net” like phenotype. Most of the reticulate mutants reported so far display green paraveinal regions and yellow interveinal regions and several reticulate mutants have been characterized at the molecular level (López-Juez et al., 1998; Yu et al., 2007). The mutation of *CHLOROPHYLL A/B BINDING PROTEIN* (*CAB*) *GENE UNDEREXPRESSED 1* (*CUE1*) in Arabidopsis leads to a reticulate phenotype (Streatfield et al., 1999). *CUE1* encodes a chloroplast envelope localized phosphoenolpyruvate (PEP)/phosphate translocator, which is responsible for transporting PEP into the chloroplast for the shikimate pathway and the synthesis of aromatic amino acids (Streatfield et al., 1999). The addition of aromatic amino acids can rescue the reticulate phenotype of *cue1* mutants, suggesting a role for aromatic amino acids in the regulation of mesophyll chloroplast development (Streatfield et al., 1999). *RETICULATA* (*RE*) is another Arabidopsis locus that leads to a reticulate phenotype when mutated and *RE* encodes a novel protein of unknown functions that is also known as LOW CELL DENSITY 1 (LCD1) (Barth and Conklin, 2003; González-Bayón et al., 2006). The Arabidopsis *differential development of vascular associated cells 1* (*dov1*) mutant defines another reticulate mutant with green paraveinal regions and yellow interveinal regions, and *DOV1* encodes glutamine phosphoribosyl pyrophosphate aminotransferase 2 (ATase2), the first enzyme in the purine nucleotide biosynthesis pathway (Kinsman and Pyke, 1998; Rosar et al., 2012). Additional Arabidopsis reticulate mutants include *venosa3* (*ven3*) and *ven6* that are defective in carbamoyl phosphate synthetase and arginine biosynthesis (reviewed in Yu et al., 2007; Mollá-Morales et al., 2011; Lundquist et al., 2014). It is interesting to note that most of the genes that are associated with the reticulate mutant phenotypes code for proteins involved in primary metabolism (Lundquist et al., 2014). In addition, reticulate mutants with yellow paraveinal regions and yellow interveinal regions are rare. The intriguing feature of these mutants implies that there are distinct tissue specific regulatory schemes controlling the differentiation of mesophyll and vascular chloroplasts.

In this work, building on our long-term efforts to identify and characterize leaf variegation mutants, we isolated two allelic *Arabidopsis thaliana* variegation mutants, designated *varigated5-1* (*var5-1*) and *var5-2*. Both mutant alleles showed a distinct reticulate leaf phenotype with yellow paraveinal regions and green interveinal regions. The molecular cloning of the *VAR5* locus revealed that *VAR5* encodes a putative magnesium transporter previously named as *AtMRS2-11*/*AtMGT10*. We confirmed that AtMRS2-11/AtMGT10/VAR5 is likely associated with the chloroplast envelope. In addition, blue native PAGE (BN-PAGE) analysis indicates that AtMRS2-11/AtMGT10/VAR5 forms large complexes in chloroplasts. Taken together, our data demonstrate that mutations in chloroplast Mg^2+^ transporter AtMRS2-11/AtMGT10/VAR5 can lead to a unique leaf reticulation phenotype and this intriguing tissue specific phenotype suggests that Mg^2+^ plays an important role in tissue specific regulation of chloroplast development.

## Materials and Methods

### Plant Materials and Growth Conditions

*Arabidopsis thaliana* wild type, *var5-1* and *var5-2* mutants (all in Columbia-0 background) seeds were grown on commercial soil mix (Pindstrup, Denmark) or on Murashige and Skoog (MS) (Caisson Laboratories, Smithfield, UT, United States) containing 1% sucrose, and maintained under continuous light (∼100 μmol⋅m^-2^s^-1^) at 22°C in controlled growth rooms. The GABI T-DNA insertion line (TAIR stock number CS323329) was obtained from the TAIR.

### Map-Based Cloning

To map the *VAR5* locus, *var5-1* was crossed with L*er* to generate the F2 mapping population. Bulked segregant analysis using a pool of DNAs from 96 F2 mapping plants with the *var5-1* phenotype and markers on all five Arabidopsis chromosomes placed *VAR5* between two molecular markers T29J13#1 and nga139 on chromosome 5. For fine mapping, 1,140 individual mapping plants were used to place the *VAR5* locus into a genomic region of 92 kb. In this region, genomic sequences of putative chloroplast genes were amplified by PCR and sequences and a point mutation in At5g22830 was detected. All primers used in this study are listed in Supplementary Table S1.

### Chlorophyll Fluorescence Measurement and Starch Staining

The maximum quantum yield of PS II (*F*_V_/*F*_M_) was measured with the Open FluorCam FC800-O system (Photon Systems Instruments, Czechia) as described (Qi et al., 2016). To reveal the starch accumulation patterns in leaves, entire rosettes were boiled in 80% ethanol (v/v) until pigments were removed, then stained with fresh iodine solution (10 g/L KI and 1 g/L I_2_) for 5 min, and de-stained in water for 1 h.

### Light Microscopy Observation of Leaf Vein

The middle main vein section from the third or fourth true leaves of wild type and *var5-1* were hand cut and fixed in 4% (v/v) glutaraldehyde in 0.1 mM PBS buffer (pH 6.8) at 4°C overnight. After fixation, samples were dehydrated in a serial dilution of ethanol and embedded in Technovit 7100 resin (Kulzer, Wehrheim, Germany). Transverse semi-thin cross sections (3 μm) were prepared with Leica RM2265 microtome, placed on glass slides, stained with fresh iodine solution. Images of iodine stained leaf cross sections were obtained with a DM5000B microscope (Leica) equipped with a CCD camera.

### Nucleic Acid Analysis and Arabidopsis Transformation

Arabidopsis DNA was isolated with the CTAB method (Yu et al., 2004). Total RNA was isolated with the TRIzol reagent according to the manufacturer’s manual (Invitrogen, Carlsbad, CA, United States). cDNAs were synthesized with the Transcriptor First Strand cDNA synthesis kit (Roche, Switzerland) with random primers. For the complementation of the *var5-1* mutant, full-length At5g22830 cDNAs were amplified with TaKaRa PrimeSTAR polymerase using primers 22830F and 22830R and subsequently cloned into the binary vector pBI111L to generate *P_35S_: At5g22830* (Yu et al., 2004). This construct was transformed in the *var5-1* mutant using the floral dip method (Clough and Bent, 1998), and transgenic plants were screened on 1/2 MS plates containing 50 mg/L kanamycin.

### Bioinformatic Analysis

AtMRS2-11/AtMGT10/VAR5 homologous proteins in different species were identified using the BLASTP program of the National Center for Biotechnology Information. Multiple sequence alignment and phylogenetic analysis were performed using the MEGA6 software (Tamura et al., 2013). Gene structures were constructed based on gene sequences retrieved from the Phytozome resource^1^ (Goodstein et al., 2012).

### Protoplast Transient Expression Assay

To construct a transient expression vector *P_35S_*:*VAR5-GFP*, full-length coding sequences of AtMRS2-11/AtMGT10/VAR5 were amplified with primers 22830GFPF and 22830GFPR and cloned into pUC18-GFP vector with the *GFP* coding sequences fused at its 3′ terminus, and the expression of VAR5-GFP was under the CaMV 35S promoter. The 35S promoter in *P_35S_*:*VAR5-GFP* was replaced by a ∼1.9 kb promoter region of *VAR5* to generate *P_V AR5_*:*VAR5-GFP*. These constructs, as well as the *P_35S_:GFP* control vector, were transformed into protoplasts isolated from 4-week-old wild-type plants as described (Yoo et al., 2007). Chlorophyll fluorescence and GFP signals were examined by a Nikon A1 confocal microscope (Nikon, Japan).

### Chloroplast Isolation, Fractionation, and BN-PAGE

Intact chloroplasts were isolated from 4-week-old Arabidopsis plants that were dark treated for 16 h (Kunst, 1998). To fractionate sub-chloroplast compartments, intact chloroplasts were broken by passing through a 24-gauge syringe in hypotonic buffer (10 mM Hepes-KOH pH7.6, 5 mM MgCl_2_). The lysate was separated on sucrose gradients by ultracentrifugation with SW41 Ti rotor at 58,000 × *g* for 2 h (Beckman Coulter L100-XP), and fractions were collected as described (Qi et al., 2016). For BN-PAGE, chloroplast samples equivalent to 10 μg of total chlorophylls were solubilized in 25BTH20G buffer [25 mM Bis-Tris-HCl, pH 7.0, 20% (w/v) glycerol] containing 1.0% (w/v) of *n*-dodecyl-β-D-maltoside (β-DM) (Järvi et al., 2011). The solubilized chloroplast samples were resolved on 3–8% BN-PAGE with a constant voltage of 70 V at 4°C, and the first dimension gel strips were denatured and resolved on 10% SDS-PAGE. For immunoblot analysis, proteins separated by SDS-PAGE were transferred onto 0.2 μm PVDF membranes with the Trans-blot Semi-Dry Transfer Cell (Bio-Rad) following the manufacturer’s manual. PVDF membranes were incubated with specific primary antibodies (Agrisera), followed by horseradish peroxidase-conjugated secondary antibodies. Signals were detected with chemiluminescence solutions and ChemiDoc Imaging System (Bio-Rad).

## Results

### The Isolation of Two Reticulate Mutant Alleles *var5-1* and *var5-2*

**FIGURE 1:**
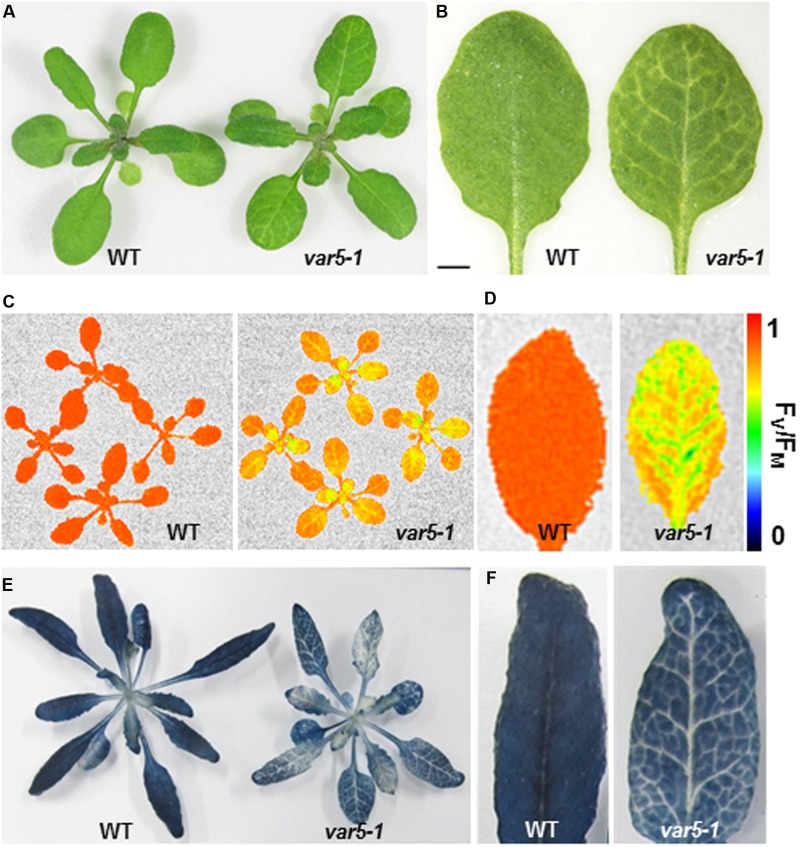
Phenotypes of the *var5-1* mutant. **(A)**. Representative 3-week-old seedlings of wild type (WT) and the *var5-1* mutant. **(B)** Details of the third or fourth true leaf from 3-week-old WT and *var5-1* mutant. **(C,D)** *F*_V_/*F*_M_ measurement of WT and the *var5-1* mutant (C for 3-week-old whole plants, and D for the detached seventh or eighth true leaves from 4-week-old plants). The color scale representing for the value of *F*_V_/*F*_M_ is at the right of the figure. **(E,F)** Iodine staining with seedlings of 4-week-old WT and the *var5-1* mutant.

During the characterization of an Arabidopsis SALK T-DNA insertion line, one unique reticulate (‘net-like’) leaf variegation mutant was recovered (**Figure 1A**; early stage of this work was carried out in the lab of Dr. Steve Rodermel at Iowa State University). A second and similar reticulate leaf variegation mutant was identified in our genetic screens for chloroplast development mutants (**Supplementary Figures S1A–D**, Qi et al., 2016). Genetic tests revealed that these two mutants were allelic and we thus designated the two mutants as *variegated5-1* (*var5-1*) and *variegated5-2* (*var5-2*), respectively, following the variegation mutant naming sequence (Martínez-Zapater, 1993; Yu et al., 2007) (**Supplementary Figures S1G,H**). Both mutant alleles showed a similar reticulate leaf variegation phenotype (**Figures 1A,B** and **Supplementary Figure S1A**). This unique leaf variegation phenotype is more pronounced in adult leaves than juvenile leaves, as the seventh true leaf of *var5-1* showed a clear reticulate phenotype, with overall yellow paraveinal regions and green interveinal regions (**Figure 1B**). Taken together, *var5-1* and *var5-2* represent two allelic mutants that display a unique leaf reticulation phenotype.

### Tissue Specific Starch Accumulation Defects in the *var5-1* Mutant

The reticulate leaf color phenotype suggests that photosynthetic capacities are compromised in *var5-1* mutants. To correlate the visible leaf color phenotype with the photosynthetic status of the mutant, we measured parameters that indicate photosynthetic performance. For the light reactions, we measured the maximum quantum yield of PS II, also known as *F*_V_/*F*_M_, using a whole plant chlorophyll fluorescence imaging system. The average *F*_V_/*F*_M_ of 15 min dark adapted wild-type whole plants was 0.82, while that of *var5-1* was 0.66, suggesting that photosynthesis is compromised in the mutant (**Figure 1C**). In addition, while a single leaf of wild-type plant showed a uniform distribution of *F*_V_/*F*_M_ values, the *F*_V_/*F*_M_ from *var5-1* also showed a ‘net-like’ pattern, with paraveinal regions showing higher value while interveinal regions having lower *F*_V_/*F*_M_ (**Figure 1D**). We next examined the accumulation of starch in the wild type and *var5-1* plants as one indicator of photosynthetic productivities. Interestingly, the starch accumulation in the *var5-1* mutant shows clearly a reticulate pattern, clearly matching both the leaf reticulation and *F*_V_/*F*_M_ patterns (**Figures 1E,F**). In addition, iodine stained leaf cross sections showed that chloroplasts in the palisade mesophyll cells on the top of vascular bundles in the *var5-1* mutant, were significantly less stained compared to that of wild type (**Supplementary Figures S2A–D**), suggesting the development of chloroplasts in the yellow paraveinal regions are compromised in the *var5-1* mutant. These data indicate that the mutation in *var5-1* leads to a unique expression of leaf reticulation and a pronounced reduction of photosynthetic capacities as indicated by PSII activities and starch accumulation in paraveinal regions than in interveinal regions.

### The Molecular Cloning of *VAR5*

**FIGURE 2:**
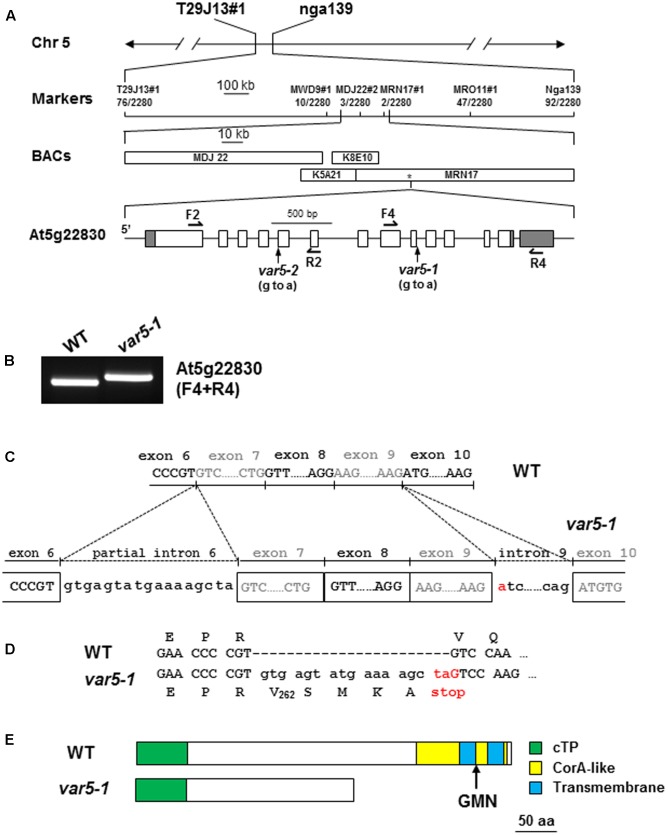
The molecular cloning of *VAR5*. **(A)** The map based cloning of *VAR5*. The mutation of *var5-1* was linked to markers T29J13#1 and nga139 on chromosome 5. A total of 1,140 F2 individual plants (2,280 chromosomes) were used in fine mapping, and the numbers of recombinants are listed under each marker. The asterisk indicates the position of At5g22830. In At5g22830 gene model, gray boxes and white boxes represent UTR and exons, respectively, and solid lines indicate introns. The position of the G to A mutation in *var5-1* was shown. **(B)** RT-PCR analysis of At5g22830 transcripts in WT and *var5-1*. Primers used in the amplifications were indicated in the parentheses. **(C)** The molecular nature of mutations in *var5-1*. The G to A transition mutation was shown red in the first nucleotide of intron 9 in At5g22830. Intron 9 and partial intron 6 remain in the At5g22830 transcripts in *var5-1*. **(D)** The *var5-1* mutation leads to a premature stop codon at the end of partial intron 6. **(E)** Based on the sequences of abnormal mRNAs of At5g22830 in *var5-1*, the putative translation products of mutant mRNA of At5g22830 was indicated. Domains are indicated with colored boxes, and cTP for chloroplast transit peptide.

Genetic analysis revealed that the reticulate phenotype of *var5-1* was likely caused by a single nuclear recessive mutation, and map-based cloning procedures were used to clone the *VAR5* locus (**Figure 2**). Initial mapping with molecular markers on all five chromosomes and subsequent fine mapping using 1,140 mapping plants placed *VAR5* in a ∼92 kb genomic region between markers MDJ22#2 and MRN17#1 on chromosome 5 (**Figure 2A**). Based on the leaf color phenotype, genomic DNA sequences of nuclear genes for chloroplast proteins in this interval were examined. A single G to A transition mutation was identified in the first nucleotide of intron 9 in At5g22830 (**Figure 2A**). At the mRNA level, amplification products spanning the mutation site appeared longer in the mutant than in wild type, suggesting pre-mRNA splicing might be affected in *var5-1* (**Figure 2B**). Sequencing of the full-length At5g22830 cDNA in *var5-1* revealed the nature of pre-mRNA splicing defects in *var5-1*. The mutation in the intron 9 led to the retention of the entire intron 9 in the At5g22830 transcript (**Figure 2C**). Surprisingly, a partial segment of intron 6 (the first 17 bp of the 328 bp wild-type intron 6) was also retained (**Figure 2C**). The translation of this aberrant mRNA would in theory give rise to a protein with the first 262 amino acids of the annotated 459 amino acids of wild-type protein plus four additional amino acids before a premature stop codon (**Figure 2D**). In theory, this early stop codon would abolish the C-terminal transmembrane domains and the conserved GMN motif (**Figure 2E**). We also determined the mutation site in *var5-2* and identified a G to A mutation at the last nucleotide of intron 4, which also caused abnormal At5g22830 transcript in *var5-2* (**Supplementary Figures S1E,F**). This mutation led to the retention of the entire intron 4 (80 bp) in the At5g22830 transcript and a putative translation product with the first 208 amino acids of wild-type protein before a premature stop codon (**Supplementary Figures S1E,F**).

**FIGURE 3:**
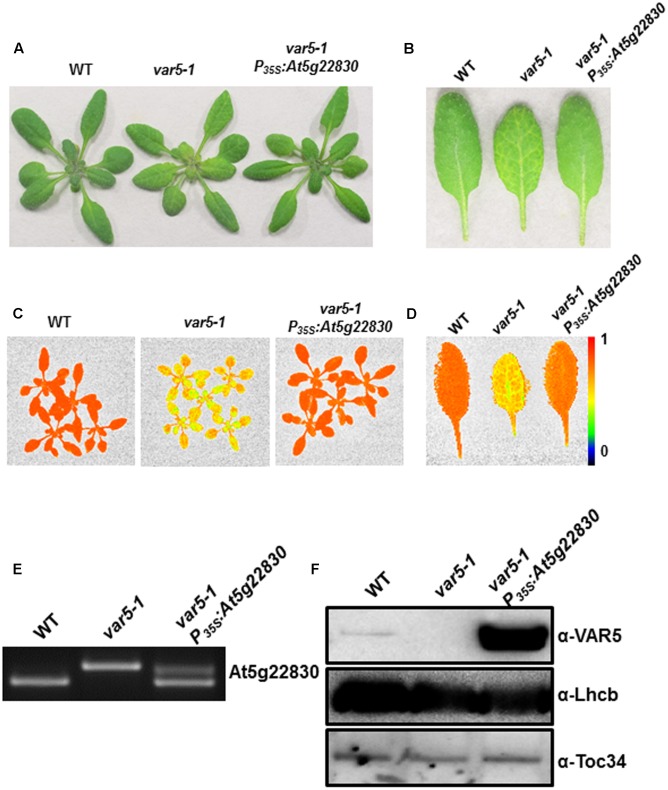
Complementation of *var5-1*. **(A)** Representative 3-week-old WT, *var5-1*, and the *var5-1* complementation line (*var5-1 P_35S_:At5g22830*). **(B)** Representative individual leaves of WT, *var5-1*, and *var5-1 P_35S_:At5g22830*. **(C,D)** *F*_V_/*F*_M_ measurement of WT, *var5-1* and *var5-1 P_35S_:At5g22830* (C for 18-day-old whole plants, and D for the detached third or fourth true leaves from 18-day-old plants). The color scale representing for the value of *F*_V_/*F*_M_ is at the right of the figure. **(E)** At5g22830 transcript accumulations in WT, *var5-1*, and *var5-1 P_35S_:At5g22830*. Total leaf RNA was extracted, and subjected to reverse transcription PCR with primers F4 and R4. **(F)** At5g22830 protein accumulations in WT, *var5-1*, and *var5-1 P_35S_:At5g22830*. Membrane fractions were isolated from leaf tissues and proteins were separated with 10% SDS-PAGE. Immunoblots were probed with antibodies against At5g22830, Lhcb and Toc34.

To confirm that the reticulate phenotype of *var5-1* was indeed caused by the mutation identified in At5g22830, a binary vector (*P_35S_: At5g22830*) containing the wild-type At5g22830 cDNA under the control of the CaMV 35S promoter was constructed and transformed into *var5-1*. Multiple transgenic lines that showed completely green leaf color and the presence of wild-type cDNA amplification products were identified, indicating that the reticulate phenotype of *var5-1* can be rescued by the wild-type At5g22830 (**Figures 3A–D**). At the mRNA level, the complementation lines showed the presence of wild-type At5g22830 cDNA (**Figure 3E**). At the protein level, we generated polyclonal antibodies against amino acid residues from 61 to 263 of At5g22830 and Western blotting analyses detected the accumulation of At5g22830 protein in the wild type, but not in the *var5-1* mutant (**Figure 3F**). In contrast, At5g22830 protein accumulation was greatly enhanced in the *var5-1 P_35S_:At5g22830* complementation line (**Figure 3F**). Similar results were obtained with *var5-2* mutant (**Supplementary Figure S1G**). The identifications of mutations sites in *var5-1* and *var5-2*, the molecular complementation and the western data show that At5g22830 is *VAR5*.

### *VAR5*/*At5g22830* Encodes a Putative CorA Family Magnesium Transporter, *AtMRS2-11*/*AtMGT10*, in Arabidopsis

**FIGURE 4:**
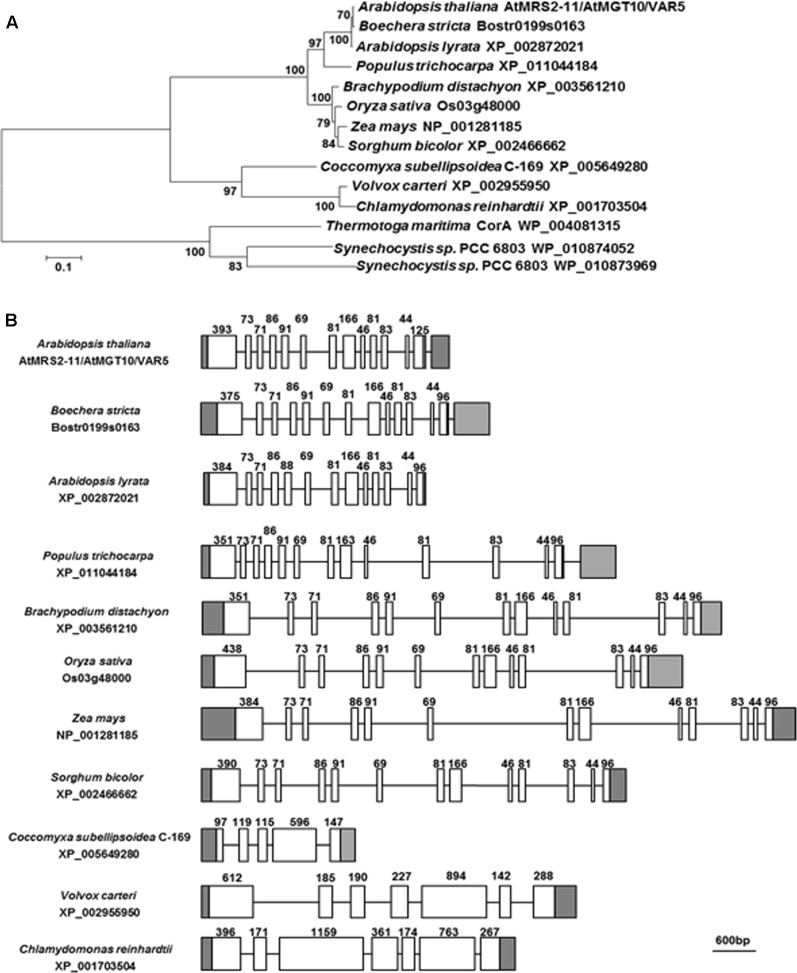
The *AtMRS2-11*/*AtMGT10*/*VAR5* gene family. **(A)** Phylogenetic analysis of the AtMRS2-11/AtMGT10/VAR5 protein family. Full-length protein sequences of AtMRS2-11/AtMGT10/VAR5 homologous proteins from dicots *Arabidopsis thaliana*, *Boechera stricta*, *Arabidopsis lyrata*, *Populus trichocarpa*, monocots *Brachypodium distachyon*, *Oryza sativa*, *Zea mays*, *Sorghum bicolor*, green algae *Coccomyxa subellipsoidea*, *Volvox carteri*, *Chlamydomonas reinhardtii*, and prokaryotes *Thermotoga maritime* and *Synechocystis* sp. PCC6803 were obtained from NCBI. The phylogenetic tree was constructed with MEGA6 (Tamura et al., 2013). The accession numbers were included. **(B)** Gene Structures of *AtMRS2-11*/*AtMGT10*/*VAR5* homologous genes. Genomic DNA sequences were retrieved from the Phytozome (https://phytozome.jgi.doe.gov/pz/portal.html). In gene structures, white boxes and lines indicate exons and introns, respectively. Gray boxes indicate UTRs. Lengths of coding exons sequences were shown.

*VAR5*/*At5g22830* was previously reported to encode a putative CorA family Mg^2+^ transporter that is also known as *AtMRS2-11*/*AtMGT10* (Schock et al., 2000; Li et al., 2001; Drummond et al., 2006). At the protein level, AtMRS2-11/AtMGT10/VAR5 contains the characteristic features of CorA Mg^2+^ transporters, including the two C-terminal transmembrane domains and in between the Mg^2+^-binding GMN motif (Lunin et al., 2006; Pfoh et al., 2012). There are at least 10 members of CorA family Mg^2+^ transporters in *Arabidopsis thaliana*, and some of them are predicted to be targeted into organelles including the chloroplast and the mitochondrion (Gebert et al., 2009). We analyzed the relationship between AtMRS2-11/AtMGT10/VAR5 and its putative chloroplast-localized homologs from various eukaryotic photosynthetic organisms, as well as CorA homologs from bacteria (**Figure 4A**). Overall, AtMRS2-11/AtMGT10/VAR5 homologs from different organisms are highly conserved (**Figure 4A**). In addition, these proteins can be grouped into major clades including eudicot, monocot, algae and prokaryotic organisms (**Figure 4A**). The evolutionary relationship between these genes were also obvious at the DNA level as putative higher plant AtMRS2-11/AtMGT10/VAR5 related genes shared identical gene structures, suggesting these genes emerged before the divergence of eudicot and monocot plants (**Figure 4B**). In contrast, AtMRS2-11/AtMGT10/VAR5 homologs from green algae did not share conserved gene structures with their plant counterparts (**Figure 4B**). These data suggest that the CorA family Mg^2+^ transporter is prevalent and highly conserved, consistent with the critical roles Mg^2+^ plays in prokaryotic and eukaryotic cells.

### AtMRS2-11/AtMGT10/VAR5 Localizes to the Chloroplast Envelope

**FIGURE 5:**
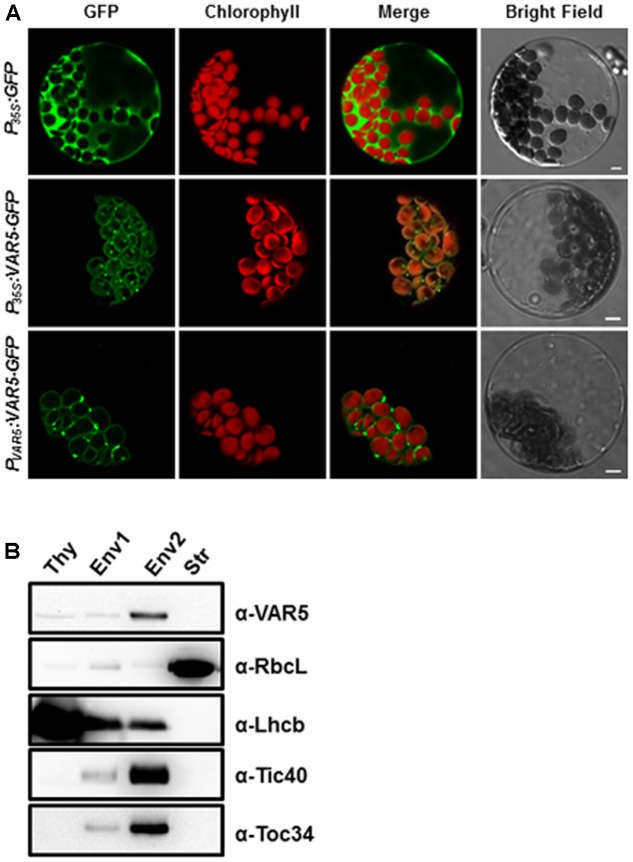
Subcellular localization of AtMRS2-11/AtMGT10/VAR5. **(A)** Protoplasts from 3-week-old WT plants were transformed with vectors containing the full-length *VAR5* (*P_35S_:VAR5-GFP* and *P_V AR5_:VAR5-GFP*) and the GFP control (*P_35S_:GFP*) and examined via confocal microcopy. Representative protoplasts with GFP fluorescence were shown. Chlorophyll is for chlorophyll fluorescence, and bars are 5 μm. **(B)** Fractions of thylakoid (Thy), Env1 (mixed envelope and thylakoids), Env2 (mainly envelope) and stroma (Str) were isolated from WT chloroplasts and subjected to immunoblot. Antibodies against RbcL (stroma control), Lhcb (thylakoid control), Tic40 and Toc34 (envelope control) were used to indicate different fractions.

AtMRS2-11/AtMGT10/VAR5 is predicted by the ChloroP program to contain a putative chloroplast transit peptide (Emanuelsson et al., 1999). Experimentally, it was shown to be localized on the periphery of chloroplasts (Drummond et al., 2006). We took two approaches to independently confirm the sub-cellular localization of AtMRS2-11/AtMGT10/VAR5. First, we used the protoplast transient expression assay to examine the localization of VAR5-GFP under the control of the 35S promoter (*P_35S_:VAR5-GFP*) and the endogenous *VAR5* promoter (*P_V AR5_:VAR5-GFP*), respectively (**Figure 5A**). Control vector (*P_35S_:GFP*) gave GFP signals mainly in the cytosol and the nucleus (**Figure 5A**). In contrast, both *P_35S_:VAR5-GFP* and *P_V AR5_:VAR5-GFP* gave GFP signals that outlined the periphery of the chloroplast, as well as some puncta inside the chloroplast, suggesting both promoters can direct VAR5 to sub-chloroplast locations that likely correspond to the chloroplast envelope (**Figure 5A**). Next, we determined the AtMRS2-11/AtMGT10/VAR5 sub-chloroplast localization biochemically (**Figure 5B**). Intact chloroplasts were fractionated into the thylakoids (Thy), the stroma (Str), the envelope fraction 1 (Env1) and 2 (Env2) with sucrose gradient centrifugations (Qi et al., 2016). Using antibodies against chloroplast proteins with known sub-chloroplast localizations, we showed that RbcL (Rubisco large subunit) was enriched in the stroma fraction, and Lhcb (PSII light harvesting complex protein) was enriched in the thylakoids fraction as expected. The enrichment of chloroplast envelope membranes in the Env2 fraction was indicated by chloroplast envelope proteins Tic40 and Toc34 (components of the Translocon at the Inner/Outer Envelope Membrane of Chloroplasts, TIC/TOC) (**Figure 5B**; Nakai, 2015). Using VAR5 antibodies that we had generated, we found that the majority of AtMRS2-11/AtMGT10/VAR5 was present in the Env2 fraction, similar to the distribution patterns of TIC/TOC components, suggesting that it is likely a chloroplast envelope membrane protein (**Figure 5B**).

### AtMRS2-11/AtMGT10/VAR5 Forms Large Protein Complexes in the Chloroplast

**FIGURE 6:**
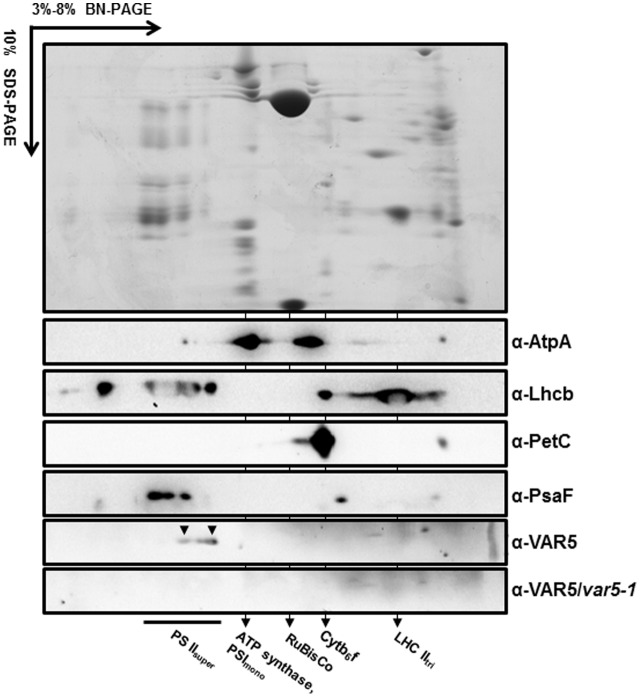
AtMRS2-11/AtMGT10/VAR5 forms large molecular weight complexes. Intact chloroplasts were solubilized with 1.0% β-DM, and subjected to first dimension 3–8% blue native PAGE (BN-PAGE). The first dimension gel strips were cut, denatured and resolved in 10% SDS-PAGE for the second dimension. Antibodies against subunits of different photosynthetic complexes were used for immunoblotting. The immunoblotting of second dimension BN-PAGE of *var5-1* sample was used for a negative control.

The crystal structure of the thermophilic bacterium *Thermotoga maritima* CorA Mg^2+^ transporter indicates that it forms a homopentamer (Lunin et al., 2006). To determine whether AtMRS2-11/AtMGT10/VAR5 forms complexes in Arabidopsis, two-dimensional BN-PAGE was carried out (Järvi et al., 2011). BN-PAGE can effectively separate chloroplast membrane protein complexes, and the estimated sizes of the major photosynthetic protein complexes are known (**Figure 6**). For instance, the sizes of the four large PSII supercomplexes were estimated to be 880–1,400 kDa (Caffarri et al., 2009; Hou et al., 2015), while the PSI monomer and the rubisco holoenzyme migrate at approximately 600 and 540 kDa, respectively (Berghöfer and Klösgen, 1999; Järvi et al., 2011). Western blot analysis with second dimension gels using antibodies against AtpA (the α subunit of the chloroplast ATP synthase), Lhcb, PetC (a subunit of the cytochrome *b*_6_*f* complex) and PsaF (a subunit of the PSI) clearly identified various photosynthetic complexes at expected sizes (**Figure 6**). Interestingly, AtMRS2-11/AtMGT10/VAR5 antibodies detected a streak of signals in the second dimension gels (**Figure 6**). Based on our results, it is clear that different forms of complexes containing AtMRS2-11/AtMGT10/VAR5 exist in the chloroplast, and the sizes of these complexes are clearly larger than the PSI monomer (∼600 kDa) (**Figure 6**). The distribution of AtMRS2-11/AtMGT10/VAR5 complexes overlapped partially with the PSII supercomplexes, which suggest the complexes could reach over 800 kDa (**Figure 6**). This is in stark contrast to the CorA complexes in *Thermotoga maritime*, which have molecular weights around 210 kDa as homopentamers (Lunin et al., 2006; Matthies et al., 2016). We did not detect AtMRS2-11/AtMGT10/VAR5 signals in *var5-1* plants, agreeing with our one dimension SDS-PAGE results (**Figure 2**). These data suggest that AtMRS2-11/AtMGT10/VAR5 forms large complexes and the complex compositions are different in Arabidopsis from those in bacteria.

## Discussion

We are interested in the genetic regulation of chloroplast development and have characterized Arabidopsis leaf variegation mutants that are results of mutations in nuclear genes (Yu et al., 2007; Liu et al., 2010; Putarjunan et al., 2013). These variegation mutants are intriguing because non-uniform leaf color phenotypes can arises from uniform genetic mutant backgrounds. Plant variegation mutants can be categorized based on their variegation patterns. The first type of variegation mutants shows apparently random distributions of chlorophyll accumulation and/or chloroplast development. Mutants in this category include *immutans* (*im*), *yellow variegated* (*var2*), and *thylakoid formation* (*thf*) (reviewed in Yu et al., 2007). The second type, the virescent mutant, displays differential chlorophyll accumulation and/or chloroplast development along the leaf proximal-distal axis, usually with the leaf base regions containing less chlorophylls and tip regions containing higher levels of chlorophylls (López-Juez et al., 1998). The third type displays differential chlorophyll accumulation and/or chloroplast development between paraveinal regions and interveinal regions, giving rise to a reticulate pattern. A number of reticulate mutants including *chlorophyll a/b binding protein (CAB) gene underexpressed 1* (*cue1*), *reticulata* (*re*; also known as *lower cell density 1*, *lcd1*), and *differential development of vascular associated cells 1*(*dov1*) have been reported and characterized (Kinsman and Pyke, 1998; Streatfield et al., 1999; Barth and Conklin, 2003; González-Bayón et al., 2006).

In this report, we identified two new allelic leaf variegation mutants in *Arabidopsis thaliana* that display a striking reticulate phenotype and designated these mutants as *var5-1* and *var5-2*. We cloned *VAR5* via map-based cloning procedures and identified mutations in At5g22830 in *var5-1* and *var5-2*, respectively. Both mutants contain mutations in the conserved sequences that form the plant intron borders and these mutations lead to the mis-splicing of pre-mRNA of At5g22830 (**Figure 2**; Lorković et al., 2000). It is worthy of noting that the mutation in intron 9 caused mis-splicing of intron 6 in *var5-1*, suggesting that downstream intron sequences can influence the splicing of upstream introns (Nesic and Maquat, 1994). Our data from two mutant alleles, the molecular cloning of *VAR5* and molecular complementation results clearly establish that the reticulate leaf phenotype of *var5-1* and *var5-2* is the result of mutations in At5g22830, which is also known as *AtMRS2-11*/*AtMGT10*.

*AtMRS2-11*/*AtMGT10*/*VAR5* encodes a CorA family of Mg^2+^ transporter in Arabidopsis. In bacteria, the canonical CorA is situated at the plasma membrane and regulates Mg^2+^ homeostasis inside the cell (Maguire, 2006). CorA family Mg^2+^ transporters are also present in higher plants (Schock et al., 2000; Li et al., 2001). In *Arabidopsis thaliana*, the 10 members of the CorA family of Mg^2+^ transporters are shown to be targeted into different sub-cellular compartments, with AtMRS2-11/AtMGT10/VAR5 as the sole member that is predicted to contain a chloroplast transit peptide (Gebert et al., 2009). Given the many processes Mg^2+^ is involved in the chloroplast, it is reasonable to assume that Mg^2+^ transporters need to be present in the chloroplast envelope to facilitate the transport of Mg^2+^. Indeed, a previous report has shown that AtMRS2-11/AtMGT10/VAR5 is possibly located at the periphery of the chloroplast through fluorescent microscopy observations (Drummond et al., 2006). In this work, we demonstrated that AtMRS2-11/AtMGT10/VAR5 is likely located to the chloroplast envelope using two approaches. First, through protoplast transient expression and confocal microscopy, we showed that VAR5-GFP can be localized to sub-chloroplast compartments that likely correspond to the chloroplast envelope (**Figure 5**). We also confirmed the chloroplast envelope localization of VAR5 biochemically and found that VAR5 showed similar localization patterns with components of the TIC/TOC complexes at the chloroplast enveloped (**Figure 5**; Nakai, 2015). To our surprise, we did observe VAR5-GFP signals inside the chloroplast with both the 35S and the endogenous *VAR5* promoter driven VAR5-GFP, and we do not yet know the nature of this phenomenon. Consistent with the chloroplast localization of AtMRS2-11/AtMGT10/VAR5, the two *VAR5* mutant alleles we identified show clear defects of chloroplast development (**Figure 1**). The reticulate phenotype of both mutants indicates a tissue specific expression of mutant phenotype. There are several possible explanations for this intriguing phenotype. It is possible that the essential roles of vascular tissues for transporting may necessitate higher levels of AtMRS2-11/AtMGT10/VAR5 activities and the lack of the transporter in *var5-1* mutants would lead to more detrimental effects in these tissues. Alternatively, other types of Mg^2+^ channels, such as fast activating chloroplast cation channels (FACC), may also be present in the chloroplast envelope to transport Mg^2+^ (Pottosin and Dobrovinskaya, 2015). It is thus possible that they may compensate for the lack of AtMRS2-11/AtMGT10/VAR5 in the interveinal regions. It was shown that AtMRS2-11/AtMGT10/VAR5 displays tissue specific expression patterns (Drummond et al., 2006; Gebert et al., 2009). However, the tissue expression pattern does not correlate with the reticulate mutant phenotype. Because both alleles we identified contain point mutations that lead to mis-regulated splicings of *VAR5* pre-mRNA, we also sought to identify a T-DNA insertion line for *VAR5*. In a previous report, no homozygous mutant plant was recovered in a GABI T-DNA insertion line (TAIR stock number CS323329), which has a T-DNA insertion in the sixth intron, suggesting that the complete loss of *AtMRS2-11*/*AtMGT10*/*VAR5* might be lethal (Gebert et al., 2009). Characterizing the same T-DNA line, we identified the precise insertion site of the T-DNA in *VAR5* but also failed to obtain homozygous insertion plants (data not shown). It is possible that the null allele of *VAR5* is lethal at early stage of embryo development. However, we could not rule out the possibility that additional secondary mutations may contribute to this lethality, as reported for other cases (Gebert et al., 2009; Bergelson et al., 2016).

In this report, we clearly demonstrated that AtMRS2-11/AtMGT10/VAR5 forms large complexes and the estimates of the molecular weights of these complexes are exceeding 800 kDa (**Figure 6**). The formation of complexes for the CorA family of Mg^2+^ transporters is not unexpected as it has been shown in bacteria that CorA forms homopentamers with a molecular weight of ∼200 kDa (Lunin et al., 2006; Matthies et al., 2016). However, the sizes we observed for AtMRS2-11/AtMGT10/VAR5 complexes are surprising and well beyond its bacterial counterparts. Although we don’t know the precise nature of these AtMRS2-11/AtMGT10/VAR5-containing complexes, it does suggest that the formation and regulation of CorA transporter complexes are different in plants, at least in the chloroplast. It is possible that in Arabidopsis chloroplasts, AtMRS2-11/AtMGT10/VAR5 forms homo-oligomers that contain more than five subunits. Alternatively, AtMRS2-11/AtMGT10/VAR5 complexes in the chloroplast may contain additional components. Considering the importance roles Mg^2+^ plays in the chloroplast, it is reasonable to assume that addition subunits may be necessary to serve structural or regulatory functions. The future determination of the components of these complexes containing AtMRS2-11/AtMGT10/VAR5 would shine more light on the functions of CorA family Mg^2+^ transporters in the chloroplast and the roles it plays in chloroplast development and biomass production, especially under low-magnesium or plus-magnesium conditions.

## Author Contributions

FY conceived and coordinated the study. SL and YQ designed, performed, and analyzed the experiments shown in **Figures 1**–**3** and supplemental data. YQ and JZ designed, performed, and analyzed the experiments shown in **Figures 4**, **6**. YL and RW designed, performed, and analyzed the experiments shown in **Figure 5**. JS, LA, and XL provided technical assistance and contributed to the preparation of the figures. FY and YQ wrote the manuscript. All authors reviewed the results and approved the final version of the manuscript.

## Conflict of Interest Statement

The authors declare that the research was conducted in the absence of any commercial or financial relationships that could be construed as a potential conflict of interest.

## Footnotes

1 http://phytozome.jgi.doe.gov/

## Supplementary Material

**FIGURE S1 |** The molecular nature of *var5-2*. **(A)** Representative 3-week-old seedlings of wild type (WT) and the *var5-2* mutant. **(B)** Details of the third or fourth true leaf from 3-week-old WT and *var5-2* mutant. **(C,D)** *F*_V_/*F*_M_ measurement of WT and the *var5-2* mutant (C for 3-week-old whole plants, and D for the detached third or fourth true leaves from 3-week-old plants). The color scale representing for the value of *F*_V_/*F*_M_ is at the right of the figure. **(E)** The molecular nature of mutations in *var5-2*. Intron 4 remains in the At5g22830 transcripts in *var5-2*. **(F)** The *var5-2* mutation leads to a premature stop codon at the beginning of intron 4. Based on the sequences of abnormal mRNAs of At5g22830 in *var5-2*, the putative translation products of mutant mRNA of At5g22830 was indicated. Domains are indicated with colored boxes. H. The representative first or second true leaf from 3-week-old WT, *var5-1*, *var5-2*, and *var5-1 var5-2* F1 generation. **(G)** The accumulation of VAR5 in WT, *var5-1*, *var5-2* and *var5-1 var5-2* F1. TIC40 was used for the envelope protein control.

**FIGURE S2 |** Light microscopy of leaf sections. The middle main vein section from the third or fourth true leaves of 2-week-old WT and *var5-1* were fixed and observed with iodine staining. **(A)** WT; **(B)** *var5-1*. **(C,D)** The dash line areas from **A** and **B** were enlarged.

## Abbreviations

AtMGT: *Arabidopsis thaliana* Mg^2+^ transporters
AtMRS2: *Arabidopsis thaliana* mitochondrial RNA splicing2
CaMV: Cauliflower Mosaic Virus
CorA: Co^2+^ resistance A
GFP: green fluorescence protein
GMN: glycine–methionine–asparagine motif
L*er*: Landsberg *erecta*
PSI: photosystem I
PSII: photosystem II, UTR: untranslated region
*VAR5*: *Variegated5*
