# Supplementary figures and images for: Mutations in the Arabidopsis AtMRS2-11/AtMGT10/VAR5 Gene Cause Leaf Reticulation
Source: Front Plant Sci. 2017 Nov 27;8:2007. doi: 10.3389/fpls.2017.02007 (PMC5712471; doi:10.3389/fpls.2017.02007)

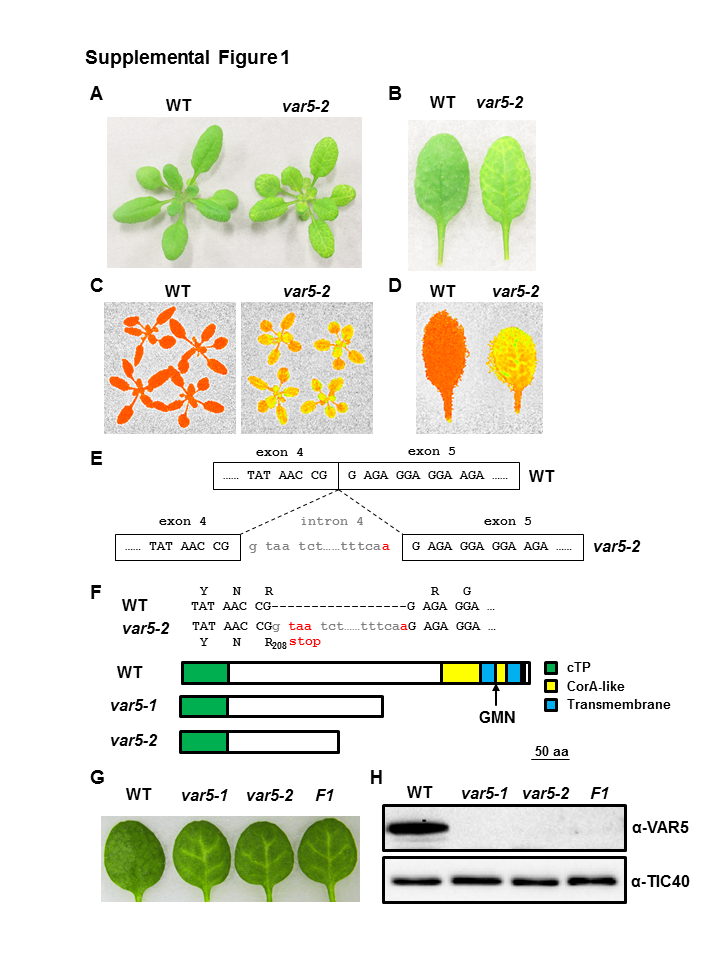

Supplement: Supplementary file 1 [file Image_1.TIF]

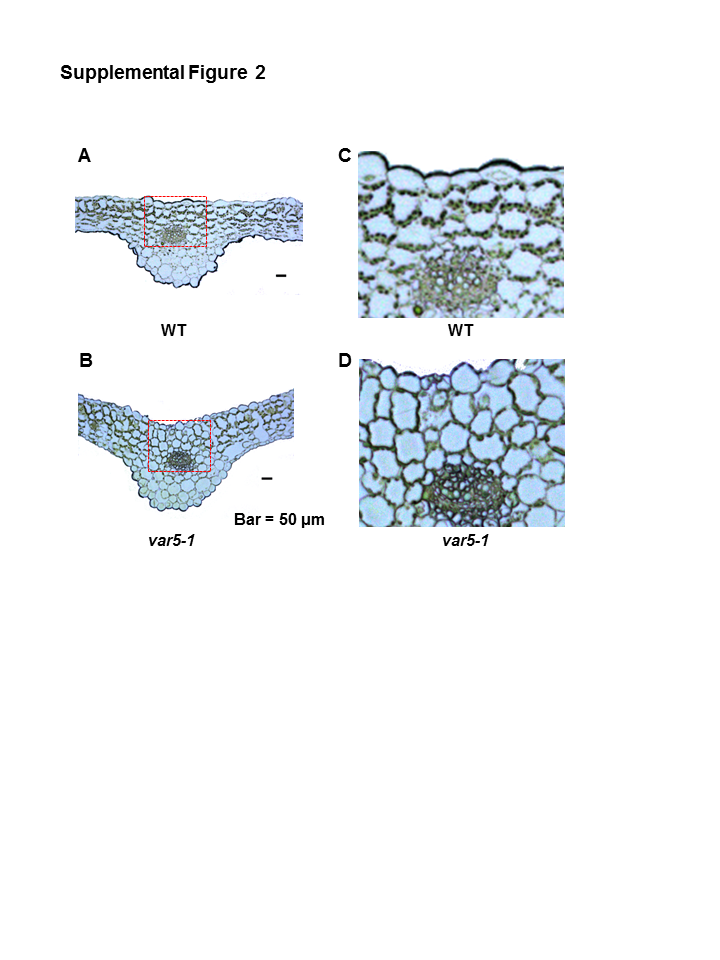

Supplement: Supplementary file 2 [file Image_2.TIF]
