# Supplementary material for: Mutations in the Arabidopsis AtMRS2-11/AtMGT10/VAR5 Gene Cause Leaf Reticulation
Source: Front Plant Sci. 2017 Nov 27;8:2007. doi: 10.3389/fpls.2017.02007 (PMC5712471; doi:10.3389/fpls.2017.02007)
Supplement: Supplementary file 3 [file Table_1.DOCX]

| Primer name | Primer sequence | Notes |  |
| --- | --- | --- | --- |
| T29J13#1F | 5'-CGC TTT AGT GAT TGT AAC TG-3' | Indel 25/-25 |  |
| T29J13#1R | 5'-TTA TCT CAC CGA CTC AAG TA-3' |  |  |
| MWD9#1F | 5'-GGA TTT GAG TAA TTG CTA GG-3' | Indel 15/-15 |  |
| MWD9#1R | 5'-TTA GAT CTG GTT GGT TCT GA-3' |  |  |
| MDJ22#2F | 5'-AGA TCC GTC AGC ATC TTC AG-3' | CAPs w/*Taq*I cut Col sequence |  |
| MDJ22#2R | 5'-TAC TGT GAA CTT TCG GAG TC-3' |  |  |
| MRN17#1F | 5'-ATC CGG AAC CTT AAC AAG AG-3' | Indel 21/-21 |  |
| MRN17#1R | 5'-CGC CTA GGA AGA TCA TAC AA-3' |  |  |
| MRO11#1F | 5'-CGA AAC AGT AGA AGG AGA TG-3' | Indel 53/-53 |  |
| MRO11#1R | 5'-TGG GAA CAC AAC CAA ACT AG-3' |  |  |
| nga139 | ([Joseph Ecker](https://www.arabidopsis.org/servlets/Community?action=view&type=person&communityID=4624),1994) | SSLP |  |
| 22830F1N | 5'-TTG AGA GTG ACA AGT CAT AC-3' | Genotyping *var5-1* and *var5-2* |  |
| 22830F1 | 5'-CGG TCA ACT ACC ATA AAT TG-3' |  |  |
| 22830R1 | 5'-TCG TCT GTT AAT CTT CCT TG-3' |  |  |
| 22830F2 | 5'-TTC GTG AAC CGG TTT ATG AG-3' |  |  |
| 22830R2 | 5'-CAA TGT CCA TAA GCC TTT GC-3' |  |  |
| 22830F3 | 5'-ACC CTC TAT GCC ATT TGA AC-3' |  |  |
| 22830R3 | 5'-TGC AGA TAG TTC TCC AAG AG-3' |  |  |
| 22830F4 | 5'-CAG ATA AGC TGA TTG CTG AG-3' |  |  |
| 22830R4 | 5'-GCC ATA ATG TAA CTG TGA TG-3' |  |  |
| 22830F | 5'-CAT CTC GAG TCT CCA TAA CCA AAG CTT CTT CG-3' | Amplifying *VAR5* cDNA | |
| 22830R | 5'-CATCTCGAGCAACATTACCAAGCAATGAGG AC-3' |  |  |
| 22830gfp F | 5'-CATGTCTAGATCTCCATAACCAAAGCTTCTTCG-3' | Generating *P35S:VAR5-GFP* | |
| 22830gfp R | 5'-CAT GTC TAG AAC CAC CAC CAC CAC CAC CAA AGA TTT TGC GTC TAC TGA GAT-3' |  |  |
| 22830 self PF | 5'-CAT GAA GCT TTG CAA ACT TCA CTT AGG CAT TGG-3' | Amplifying the *VAR5* promoter region | |
| 22830 self PR | 5'-CAT GTC TAG AGC TTT GGT TAT GGA GAT TTT TAG-3' |  |  |
| 22830 EEC *Bam*HI F | 5'-CAT GGA TCC TGT TTC GCC AAG TCT CCG ACA AC-3' | Generating VAR5 antibody | |
| 22830 EEC *Hin*dIII R | 5'-CAT AAG CTT CTA GAC ACG GGG TTC AAT GTC CA-3' |  |  |

Supplementary Table S1, Primers used in this study.
